# The Reduced Genome of the *Francisella tularensis* Live Vaccine Strain (LVS) Encodes Two Iron Acquisition Systems Essential for Optimal Growth and Virulence

**DOI:** 10.1371/journal.pone.0093558

**Published:** 2014-04-02

**Authors:** Natalie Marie Pérez, Girija Ramakrishnan

**Affiliations:** 1 Department of Microbiology, Immunology and Cancer Biology, University of Virginia, Charlottesville, Virginia, United States of America; 2 Department of Medicine, Division of Infectious Disease and International Health, University of Virginia, Charlottesville, Virginia, United States of America; Université Paris Descartes, INSERM, France

## Abstract

Bacterial pathogens require multiple iron-specific acquisition systems for survival within the iron-limiting environment of the host. *Francisella tularensis* is a virulent intracellular pathogen that can replicate in multiple cell-types. To study the interrelationship of iron acquisition capability and virulence potential of this organism, we generated single and double deletion mutants within the ferrous iron (*feo*) and ferric-siderophore (*fsl*) uptake systems of the live vaccine strain (LVS). The Feo system was disrupted by a partial deletion of the *feoB* gene (Δ*feoB*′), which led to a growth defect on iron-limited modified Muller Hinton agar plates. ^55^Fe uptake assays verified that the Δ*feoB*′ mutant had lost the capacity for ferrous iron uptake but was still competent for ^55^Fe-siderophore-mediated ferric iron acquisition. Neither the Δ*feoB*′ nor the siderophore-deficient Δ*fslA* mutant was defective for replication within J774A.1 murine macrophage-like cells, thus demonstrating the ability of LVS to survive using either ferrous or ferric sources of intracellular iron. A LVS Δ*fslA* Δ*feoB*′ mutant defective for both ferrous iron uptake and siderophore production was isolated in the presence of exogenous *F. tularensis* siderophore. In contrast to the single deletion mutants, the Δ*fslA* Δ*feoB*′ mutant was unable to replicate within J774A.1 cells and was attenuated in virulence following intraperitoneal infection of C57BL/6 mice. These studies demonstrate that the siderophore and *feoB*-mediated ferrous uptake systems are the only significant iron acquisition systems in LVS and that they operate independently. While one system can compensate for loss of the other, both are required for optimal growth and virulence.

## Introduction

Iron is an essential element for most organisms, and plays a critical role in enzymatic catalysis, electron transfer, and amino acid and DNA synthesis. Nevertheless, regulation of intracellular iron levels is important for limiting the production of hydroxyl radicals that can potentially disrupt cellular membranes and DNA integrity [1]. Iron can alternate between two oxidation states, the poorly soluble ferric (Fe^3+^) form and the more soluble ferrous (Fe^2+^) forms. Within the mammalian host, iron is largely sequestered and bound by host proteins such as heme, ferritin, and transferrin, but is also available in small amounts within the labile iron pool (LIP) of the cytoplasm [2]–[4]. In this iron-limiting intracellular environment, bacterial pathogens acquire iron through the use of specific acquisition systems that are characteristically regulated by the ferric uptake regulator, Fur [5], [6]. Bacteria typically express multiple mechanisms to acquire oxidized ferric and reduced ferrous forms of iron which contribute to bacterial growth, survival, and virulence. While specific iron acquisition mechanisms are not necessarily indicators of pathogenicity, the ability to acquire iron within the host can be a determinant of bacterial virulence potential [7].

*Francisella tularensis* is a Gram-negative, facultative intracellular pathogen and the causative agent of the disease tularemia [8]. The subspecies *tularensis* and *holarctica* vary in their virulence potential, with the *tularensis* subspecies classified as a Tier One Select Agent due to its high virulence in humans (infectious dose <10). The live vaccine strain (LVS), an attenuated *holarctica* derivative is able to provide partial protection from infection by more virulent strains [8] but remains highly virulent in the mouse following intranasal or intraperitoneal infection [9]. Like the virulent strain Schu S4 belonging to subspecies *tularensis*, LVS is capable of entering and replicating in a variety of cell types, and of these, the macrophage is critical in dissemination of *F. tularensis* and is the best-studied model for intracellular replication [10]. Upon phagocytosis by a macrophage, *F. tularensis* is initially sequestered in a phagosome [11]. The bacteria escape the phagosome before fusion with lysosomes can occur [12] and enter the cytosol where they grow and replicate. The ability of *F. tularensis* to replicate within macrophages is dependent on the availability of intracellular iron as shown by inhibition of this process by deferoxamine [13].

Under iron limitation, various strains of *F. tularensis* (LVS, Schu S4) and the related *F. novicida* (U112) are capable of secreting a siderophore to acquire ferric iron from the environment [14], [15]. The genes required for biosynthesis and transport of the siderophore are located within the *fur-fslABCDE* locus (also designated as the *fur-figABCDE* locus). The *fslA* gene, which encodes a putative siderophore synthetase, is required for siderophore production in the *F. tularensis* strains LVS and Schu S4 [14], [16] and in the *F. novicida* strain U112 [15]. While siderophore production under iron-limitation is similar amongst the strains, mechanisms for siderophore utilization may differ. In Schu S4 and strain U112, the outer membrane protein FslE is essential for ferric-siderophore utilization, whereas in LVS, FslE is only partially responsible for this process [17]–[19]. Deletion of genes for siderophore biosynthesis or utilization in *F. tularensis* strain Schu S4 does not reduce virulence of the bacteria in mice [16], [20]. These results are in contrast to some other virulent intracellular pathogens such as *Bacillus anthracis* [21] and *Mycobacterium tuberculosis* [22], [23] where deletion of genes required for siderophore biosynthesis render the bacteria defective for intracellular growth within macrophages and leads to attenuated virulence in animal models of infection.

In addition to the ferric-siderophore system, the *F. tularensis* genome also contains genes governing ferrous iron uptake across both the outer and inner membranes. In the enterobacterial systems, porins are believed to allow entry of ferrous iron across the outer membrane. General porins have not been identified in *F. tularensis*, but in Schu S4 ^55^Fe transport assays demonstrated that a specific outer membrane protein FupA is required for uptake of ferrous iron at low concentrations (corresponding to levels limiting for growth in defined media) [20]. The *fupA* mutant however retained ferrous iron transport capability at higher iron concentrations indicating that FupA is specifically associated with high affinity ferrous iron transport across the outer membrane [20]. FupA is also a virulence factor, and a Schu S4 Δ*fupA* mutant is attenuated for virulence in the mouse model of infection [16], [20]. In LVS, the FupA ortholog is encoded by a hybrid gene *fupA/B* due to a recombination event that has a major attenuating effect on virulence of the strain [24], [25]. FupA/B in LVS was recently shown to be necessary both for ferrous iron uptake at low concentrations (“high affinity uptake”) and for optimal ferric-siderophore uptake, a feature that may be unique to this strain [19].

*Francisella* genomes also encode the ubiquitous Feo inner membrane ferrous iron transport system that has been best studied in the enteric Gram-negative bacterial systems *Escherichia coli* and *Salmonella enterica* serovar Typhimurium (*S*. Typhimurium) [26]–[29]. Genes encoding the Feo system are present in many bacterial pathogens including *Helicobacter pylori* [30], *Legionella pneumophila* [31], *Campylobacter jejuni* [32], *Yersinia pestis* [33], *Salmonella enterica* [34], [35], and *Shigella flexneri* [36]. The Feo system classically contains three genes encoded in the locus *feoABC* [27]. Ferrous iron is transported across the cytoplasmic membrane by the GTP-dependent permease, FeoB [37], [27]. FeoB is composed of a hydrophilic N-terminal G-protein domain and a C-terminal integral membrane domain predicted to have 8-transmembrane α-helices [37], [27] and its function is dependent on FeoA and/or FeoC [27]–[29]. In some bacterial systems, *feoA* and *feoC* genes may be located in different regions of the bacterial chromosome and the *feoC* gene may even be absent [27]. The *F. tularensis* strain LVS only encodes the *feoA* (*FTL_0660*) and *feoB* (*FTL_0133*) orthologs, which are unlinked and map to different regions of the *F. tularensis* chromosome (NCBI Reference sequence NC_007880.1).

A signature-tagged mutagenesis screen of a transposon mutant library revealed that *fslA* and *feoB* were each individually required for mouse lung infection by LVS [38]. A LVS Δ*feoB* mutant was also recently reported to have reduced virulence in an intranasal mouse infection model [39]. In our current study, we devised a strategy to generate a LVS double deletion mutant in *feoB* and *fslA*. We analyzed single and double deletion mutants to establish the primary importance of the *feoB* gene for ferrous iron uptake in LVS and to evaluate the contributions of ferrous and ferric-siderophore iron uptake to intracellular survival and virulence of *F. tularensis* strain LVS. Our studies also demonstrate conclusively that these are the only two significant iron-acquisition systems in the organism and highlight the reduced and minimalist nature of the iron uptake machinery for LVS survival and virulence.

## Materials and Methods

### Ethics statement

All mouse protocols were performed with the approval of the Animal Care and Use Committee (ACUC) of the University of Virginia (protocol #3512). The University's Animal Welfare Assurance number is #A3245-01, and the vivarium is accredited by the Association for Assessment Accreditation of Laboratory Animal Care International.

### Bacterial Strains and media

*Francisella tularensis* subspecies *holarctica*, live vaccine strain (LVS) was obtained from K. Elkins (CBER). The bacteria were maintained at 37°C on modified Muller Hinton agar, MHA (Difco), supplemented with 2.5% horse serum, 1% glucose, 0.1% cysteine, and 0.025% ferric pyrophosphate (FePPi). For the purpose of this study, iron rich MHA plates were supplemented with FePPi and annotated as MHA+. Iron-limiting MHA plates were not supplemented with iron (MHA-) but contained an undetermined amount of iron from horse serum and agar. *F. tularensis* strains were grown in liquid Chamberlin's defined media (CDM) [40] at 37°C with shaking. Bacterial optical densities were read at 600 nm (OD_600_) using a plate reader (BioTek ELx800). For growth comparisons in liquid, we used tryptic soy broth (TSB/c) supplemented with 0.1% cysteine, 0.1% glucose, and 0.025% ferric pyrophosphate (FePPi) and chelex-100 (BioRad) treated CDM (che-CDM) [14] supplemented with MgSO_4_ (0.55 mM), CaCl_2_ (5 μM) and FePPi to make the medium either iron replete (2.5 μg/mL, 3.36 μM) or iron limiting (0.125 μg/mL, 0.168 μM) [14], [20]. Bacteria in the exponential stage of growth were inoculated to an OD_600_ of 0.01 in the respective liquid growth media. For growth in iron-limiting and iron-replete che-CDM, the bacteria were first washed three times in che-CDM without iron. To maintain the LVS Δ*fslA* Δ*feoB*′ mutant, *F. tularensis* siderophore-active culture supernatant (determined by the Chrome Azurol S assay as described below in “Detection of *F. tularensis* siderophore”) was obtained from LVS cultures grown in iron limiting che-CDM liquid and 100 μL of this filter sterilized (0.22 μm) supernatant was topically added to MHA+ agar plates. A *F. tularensis* siderophore stock was obtained from siderophore-active supernatants of LVS by chromatography on AG1X-8 columns (as described in [14]). Column eluates were lyophilized and dissolved in water. A final concentration of 1.5 mM was determined by a Cu-CAS assay [41] as previously described [20]. This siderophore stock was used in some experiments to promote growth of the LVS Δ*fslA* Δ*feoB*′ mutant. For complementation studies, kanamycin was added at 15 μg/ml. Bacterial stocks used for *in vitro* assays, intracellular replication and mouse infection studies were stored at −80°C. *Escherichia coli* strain MC1061.1 (araD139 Δ(ara-leu)7696 galE15 galK16 Δ(lac)X74 rpsL(Str^r^) hsdR2 (rK^−^mK^+^) mcrA mcrB1 recA) was received from Chang Hahn (University of Virginia) and used for cloning purposes [14]. *E. coli* bacteria were grown in Luria broth (LB) and on agar plates supplemented with ampicillin, 50 μg/ml and 100 μg/ml respectively.

### Growth of serial dilutions on plates

Bacterial strains were routinely grown overnight in CDM liquid at 37°C with shaking. In experiments involving the LVS Δ*fslA* Δ*feoB*′ mutant, bacteria were grown overnight on a MHA+ plate and directly inoculated into CDM liquid on the day of the experiment. Bacterial cultures were adjusted to an OD_600_ of 1 and ten-fold serially diluted in CDM to the 10^−6^ dilution. A multichannel pipette was used to spot 5 μL of each dilution onto MHA+ or MHA- plates. Growth was assessed after 3 days at 37°C under normal aerobic conditions.

### Detection of *F. tularensis* siderophore

The liquid Chrome Azurol S (CAS) assay developed by Schwyn and Neilands 1987 [42] was adapted to assess the production of siderophore (as detailed in [14]). Bacterial strains were grown in CDM overnight to mid-logarithmic phase. Bacteria were then washed in che-CDM without iron and inoculated into che-CDM supplemented with low concentration of FePPi (0.125 μg/mL). At mid-logarithmic growth, bacteria were centrifuged at 9,000×g. Supernatant (100 μL) was collected and added to equal parts CAS solution (100 μL) and 2 μL of shuttle solution in wells of a 96-well plate. After a thirty-minute incubation at room temperature, absorbance was read at 630 nm (A_630_) on a plate reader (BioTek ELx800). CAS activity was calculated using water as a reference blank with the formula ((A_630_ water-A_630_ sample)/ A_630_ water). CAS activity was normalized to bacterial cell density (OD_600_) to obtain Specific CAS activity (CAS activity/OD_600_). All strains were tested in triplicate.

### Construction of LVS Δ*feoB*′ and LVS Δ*fslA* Δ*feoB*′ strains and complements

A partial deletion mutant of the *feoB* gene (Δ*feoB*′) was generated through the use of a suicide vector in a two-step mutagenesis procedure [17]. The 3′ flanking sequence corresponding to the last ten codons and 1.828 kb downstream of the stop codon was amplified using primers 5′ CTACTGGCGGCCGCTTCGTGGCAAATCTTACTGG 3′ and 5′ CTACTGGAGCTCGTAGCATGAAAAGCTTACC 3′ and was cloned as a *Not*I-*Sac*I fragment in plasmid pGIR459 [43]. The 5′ homologous sequence consisting of 480 bp upstream of the *feoB* start codon and 1.066 kB of the amino-terminal coding sequence was obtained by PCR amplification using *Pfu* DNA polymerase (Stratagene) and primers 5′CTACTGTCTAGAAGCCAATCCAAGATATGGTG 3′ and 5′ CAATTAACGGTACAAAAGCTTTGC 3′. The 5′ sequence was cloned as an *Xba*I-*Hind*III fragment with a 100 bp *Hind*III-*Not*I linker sequence derived from pCK155 [44] to generate the Δ*feoB*′ suicide plasmid pGIR473. Plasmid pGIR473 was introduced into LVS by electroporation as previously described [14] and kanamycin resistant colonies were screened by PCR to confirm integration of the plasmid in the chromosome. Integrants were plated on sucrose plates without kanamycin and colonies arising from this were screened for loss of plasmid sequences and for presence of the deletion by PCR analysis of genomic DNA.

LVS Δ*feoB*′ was used as the parental strain with the suicide plasmid pGIR457 [14] to generate LVS Δ*fslA* Δ*feoB*′ bearing an in-frame *fslA* deletion in addition to the *feoB*′ deletion. Potential Δ*fslA* Δ*feoB*′ colonies were isolated initially on MHA+ plates and were found to contain a mixed population of the parental LVS Δ*feoB*′ and LVS Δ*fslA* Δ*feoB*′ cells by diagnostic PCR. The LVS Δ*fslA* Δ*feoB*′ strain was ultimately isolated in pure culture by supplementing plates with purified LVS siderophore applied topically to the solidified agar.

The Δ*feoB*′ and Δ*fslA* Δ*feoB*′ mutants were complemented in *cis* with *feoB* by integrating a suicide plasmid bearing a kanamycin cassette at the *feoB*′ locus on the chromosome. The integrative plasmid carried a wild-type copy of *feoB* under control of the promoter of *fslA*. The *feoB* gene was amplified with primers 5′ CTACTGTCCGGAGCCAATCCAAGATATGGTG 3′ and 5′ CTACTGCATATGATTCAAATTAGAATTTTAAGAGC 3′ using Fast Start High Fidelity polymerase (Roche Applied Science). The PCR fragment was digested with *Nde*I and *BspE*I and ligated into the corresponding sites in plasmid pGIR463 [17] to generate the in *cis* complementing plasmid pGIR463_*feoB* (p*feoB*). Plasmids were introduced by electroporation and selection with kanamycin as previously described [14]. Complements were confirmed by PCR analysis of isolated DNA from the bacteria.

### Intracellular Replication Assay

Bacterial intracellular replication was assessed in murine macrophage like cells J774A.1 (ATCC TIB-67) as previously described [43]. J774A.1 cells were maintained in high glucose Dulbecco's modified Eagle's medium (DMEM) supplemented with 10% FBS and grown at 37°C with 5% CO_2_ and split 1∶10 per passage. J774A.1 cells were counted on an automated cell counter (BioRad TC 10) and seeded at a concentration of 2×10^5^ cells per well in 24-well plates the day before the assay. Bacteria were added at a multiplicity of infection (MOI) of 15 or 5 into four wells per group and plates were centrifuged at 950×g for 10 minutes at room temperature to promote the bacterial invasion process. Cells were incubated at 37°C for one hour, washed twice with PBS and incubated with 50 μg/mL of gentamicin in DMEM+FBS for 1 hour at 37°C. At two hours post-infection, wells were washed twice with PBS and cells in one set of wells lysed with distilled water and vigorous pipetting. Fresh media was added to remaining wells and incubation at 37°C continued. Lysates were prepared at 2, 24, and 48 hours and were serially diluted in CDM and plated on MHA plates to determine intracellular bacterial numbers as colony-forming units (CFU). Concentrated *F. tularensis* siderophore was additionally topically spread on MHA+ agar to promote growth of the double deletion mutant at each time point. Intracellular replication assays were repeated three times to ensure consistency in results.

### ^55^Fe uptake assays

^55^Fe uptake assays were accomplished as previously described [20], [19]. For the initial ^55^Fe uptake studies with LVS, Δ*feoB*′, and Δ*fslE* mutants, were grown overnight in iron-limiting che-CDM. For studies involving LVS Δ*fslA* Δ*feoB*′, all strains were grown overnight on MHA+ plates with topical supplementation of *F. tularensis* siderophore for the Δ*fslA* Δ*feoB*′ mutant strain. Bacteria collected from these plates were washed once in che-CDM and then inoculated in che-CDM containing no iron, followed by incubation at 37°C with shaking for 3 hours to induce expression of iron acquisition systems. Cell pellets were brought to an OD_600_ of 0.2 and 0.1 mL of the suspensions were added to an equal volume of che-CDM in 96 well filter plates (Millipore). For ^55^Fe^2+^ (ferrous iron) uptake studies, the final transport assay contained ^55^FeCl_3_ (PerkinElmer Life Sciences; 21.95 mCi/mg, 38.59 mCi/mL) at concentrations of 0.1 μM (high affinity transport) or 3 μM (low affinity uptake) in the presence of 5 mM ascorbate. For assessing ^55^Fe^3+^-siderophore uptake, the transport reaction contained 1.5 μM ^55^Fe^3+^ complexed to siderophore in the presence of 10 mM citrate. Uptake was initiated by addition of ^55^Fe to bacteria in filter wells, and accumulation was assessed at 5 and 10 minutes by scintillation counting of filtered cells. Bacterial protein content was analyzed by BCA assay (Pierce). All strains were tested in either triplicate or quadruplicate and rates of transport were normalized to protein concentration (pmol/min/mg). Transport assays were repeated three times to ensure consistency in results.

### Western Blotting

LVS and Δ*fslA*, Δ*feoB*′, and Δ*fslA* Δ*feoB*′ mutant strains were grown overnight on MHA+ plates at 37°C and resuspended in che-CDM. For comparison to growth under iron-limitation, LVS was also grown in iron limiting che-CDM liquid overnight. Bacteria were normalized to cell density (OD_600_) and lysed in 1X SDS page loading dye. Lysates were separated on 10% SDS-PAGE gels and protein was transferred onto polyvinylidene difluoride (PVDF) at 100 V. The PVDF membrane was incubated with primary and HRP-conjugated secondary antibodies for detection by chemiluminesence. The FslE peptide antibody [43] was used at a dilution of 1∶2,500 and secondary goat anti-rabbit-peroxidase conjugate antibody (Sigma) at 1∶10,000. GroEL expression was used as a loading control and was detected by the rabbit primary antibody GroEL(Sigma) was used at 1∶10,000 dilution and the secondary goat anti-rabbit-peroxidase conjugate antibody (Sigma) at 1∶10,000. FupA/B expression was detected with the FupA peptide antibody at 1∶100,000 dilution [20] and the secondary anti-guinea pig in goat 1∶50,000 (Sigma).

### Mouse infection

Frozen stocks of LVS strains were diluted in 0.9% sterile saline solution to 10,000 CFU/mL and 100 μL (1000 CFU) was injected by intraperitoneal (IP) route into seven-week-old C57BL/6 male mice (five mice per group) (Jackson laboratories, Bar Harbor, ME) [43]. The CFUs administered were determined by plating bacterial dilutions on MHA+ plates, topically supplemented with *F. tularensis* siderophore in order to promote growth of LVS Δ*fslA* Δ*feoB*′ strain. Mice were observed each day for symptoms of disease and mice were euthanized at a humane endpoint if symptoms of irreversible morbidity were observed. Survivors were subsequently challenged by IP delivery with 1000 CFU of LVS and monitored for a period of 14 days.

### Statistical Analysis

Data were analyzed using Prism 4.0 software (GraphPad Software, Inc., San Diego, CA). Statistical comparison of values was accomplished using *t* test function and the Logrank Test function was used to evaluate mouse survival curves.

## Results

### Characterization of the LVS Δ*feoB*′ mutant

To characterize the role of the Feo system in LVS iron transport, we generated a mutant LVS Δ*feoB*′ strain with an 1100 bp deletion in the 2241 bp *feoB* gene using a two-step mutagenesis procedure. The Δ*feoB*′ strain is predicted to produce a 380 amino acid truncated protein lacking 6 of the 8 predicted C-terminal transmembrane sequences, including two of the four Gate regions that constitute the permease in the inner membrane [27].

#### i. The mutant *ΔfeoB*′ strain is defective for growth on MHA+ and MHA- agar plates

**Figure 1:**
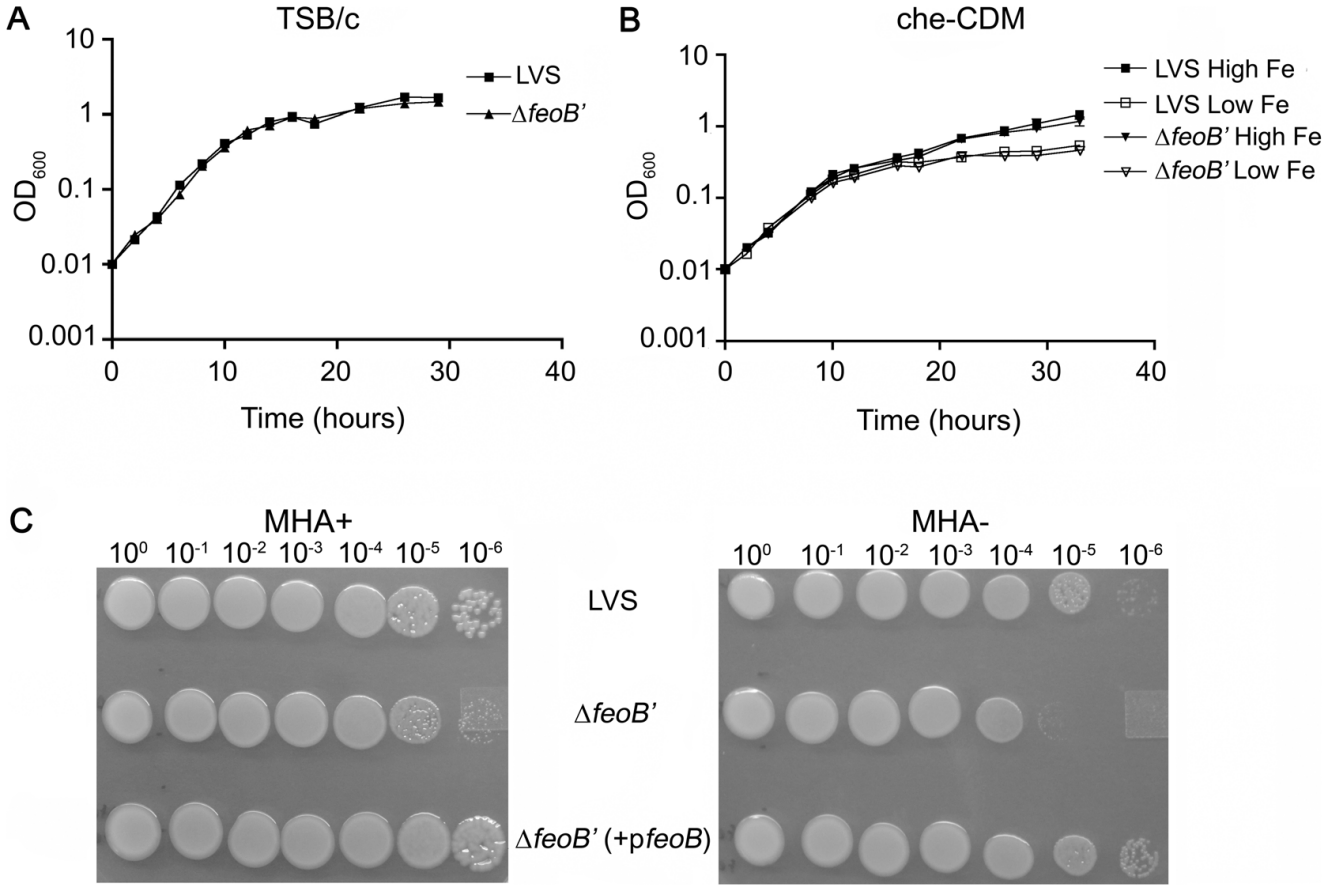
Growth of LVS Δ*feoB*′ in liquid and on agar. Bacteria were inoculated in either iron rich TSB/c (A) or in che-CDM supplemented with FePPi at 2.5 μg/mL (High Fe) or 0.125 μg/mL (Low Fe)(B) and growth was followed as change in optical density at 600 nm (OD_600_). Values were plotted as the means ± S.E. In Figure 1 C, bacterial strains were ten-fold serially diluted in che-CDM, spotted on MHA agar with (MHA+) and without (MHA-) iron supplementation and grown for 3 days at 37°C under aerobic conditions.

We tested growth of the wild- type LVS and LVS Δ*feoB*′ strains in rich broth (TSB/c) as well as in iron-replete and iron limiting minimal liquid medium (che-CDM), but did not observe growth differences between the strains (Figure 1A and 1B). The similar growth of the two strains demonstrated that expression of a truncated FeoB protein did not have deleterious effects on general growth of the mutant. We then tested growth of ten-fold serial dilutions of each strain on modified Muller Hinton (MHA+) agar plates that are iron-replete and commonly used for non-selective maintenance and propagation of *F. tularensis* strains. The Δ*feoB*′ mutant strain was able to form single colonies on these plates (Figure 1C, MHA+), but had a delayed growth phenotype. On plates lacking iron supplementation (MHA-), wild-type LVS also demonstrated a growth delay but was able to form single colonies (out to 10^-6^ dilution) while the Δ*feoB*′ mutant demonstrated a growth defect and was only able to grow out to 10^-5^ dilution compared to wild-type LVS. The observed colony size of the Δ*feoB*′ mutant was small in comparison to wild-type LVS (MHA+) and this “small” colony phenotype was further accentuated on MHA- plates. Figure 1C also shows that the reduced size and growth defect phenotype of the Δ*feoB*′ mutant was abrogated in the Δ*feoB*′ *cis-*complemented strain, Δ*feoB*′ (*+*p*feoB*). These results suggested that the observed growth delay of the Δ*feoB*′ mutant on agar was likely due to a deficiency in iron acquisition. Small colony morphology associated with reduced iron acquisition has been previously reported in LVS as well as in *E. coli feo* mutants [39], [26]. The discrepancy in liquid and agar growth phenotypes may be explained by the fact that the iron is likely in the oxidized ferric form during growth with shaking in liquid, while the agar medium with a high concentration of cysteine (required supplement since *F. tularensis* is a cysteine auxotroph) would maintain much of the iron in the reduced ferrous form.

#### ii. FeoB is required for high and low affinity iron transport

**Figure 2:**
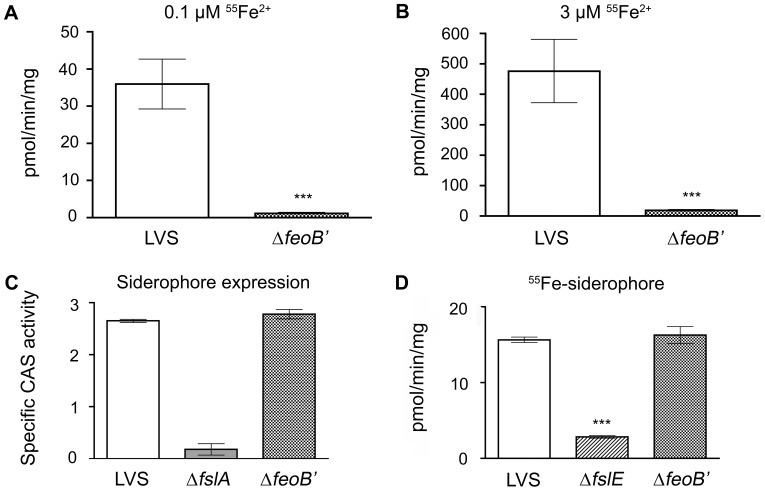
LVS Δ*feoB*′ is defective for ferrous uptake but is capable of siderophore production and utilization. A and B, ^55^Fe ferrous iron uptake. LVS and Δ*feoB*′ strains were grown overnight to mid-logarithmic phase in iron-limiting liquid che-CDM and rates of ferrous iron uptake were determined at 0.1 and 3 μM [^55^Fe^2+^] in the presence of ascorbate. C, Siderophore production. Culture supernatants of LVS, Δ*fslA*, Δ*feoB*′ strains grown overnight under iron-limitation were tested in the CAS assay and siderophore activity was normalized to OD_600_. D, Siderophore-mediated ^55^Fe uptake. ^55^Fe-bound siderophore was incubated with bacteria grown in iron-limiting che-CDM and the rate of ^55^Fe uptake was determined. Values were expressed as the means ± S.D. Significance was calculated relative to LVS values. ***p<0.001.

**Figure 3:**
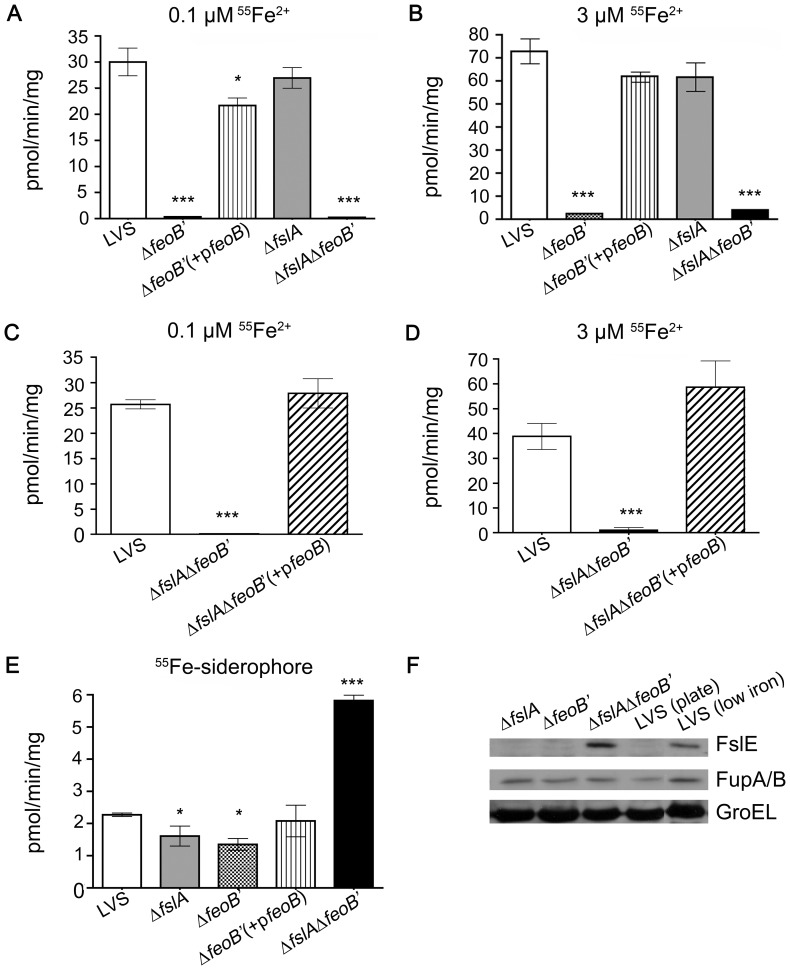
Iron acquisition by LVS single and double mutants and complementing strains. LVS, the Δ*fslA* and Δ*feoB*′ mutants and in *cis* complements as indicated were grown overnight on fresh MHA+ plates (with additional siderophore supplementation to the Δ*fslA* Δ*feoB*′ mutant) at 37°C and further inoculated into che-CDM without FePPi for three hours with shaking. Single mutants and the Δ*feoB*′ complement were assessed in A and B, and the complement of the double deletion mutant was tested in C and D. The rate of ^55^Fe uptake was determined at (A,C) 0.1 and (B,D) 3 μM [^55^Fe^2+^] in the presence of ascorbate. E, Strains were incubated with ^55^Fe bound siderophore and rate of ^55^Fe uptake was determined. F, Western blotting to determine expression levels of FslE, FupA/B, and the loading control GroEL in bacteria grown on MHA+ plates in comparison to LVS grown under iron-limiting conditions. In A–E, values are expressed as the means ± S.D. Significance was calculated relative to LVS values. * p<0.05, ***p<0.001.

^55^Fe uptake assays are the best platform to demonstrate ferrous iron transport in bacteria and have been used to establish that *F. tularensis* employs distinct outer membrane transport mechanisms for ferrous iron transport at limiting iron concentrations (high affinity transport) and at high iron concentrations (low affinity transport) [20], [19]. We compared the ability of the Δ*feoB*′ and wild-type LVS strains to take up ^55^Fe^2+^ at two iron concentrations, 0.1 μM for high affinity iron acquisition and 3 μM reflecting low affinity transport [19]. Bacterial strains were grown overnight under iron-limitation and were then incubated with ^55^Fe in the presence of ascorbate to keep the iron in the reduced form. As shown in Figure 2A and 3A, the Δ*feoB*′ strain showed a negligible rate of ferrous uptake compared to wild-type LVS at 0.1 μM iron. Surprisingly, ferrous iron transport in the Δ*feoB*′ mutant was also minimal at the 3 μM iron concentration (Figure 2B and 3B). When wild-type *feoB* was restored to the mutant, the complemented strain (Δ*feoB*′ (+p*feoB*)) was able to regain ferrous iron acquisition capability at both low and high concentrations (Figure 3). These results indicated that *F. tularensis* strain LVS relies solely on FeoB for ferrous iron acquisition across the inner membrane.

#### iii. FeoB is not required for siderophore production or siderophore-mediated iron acquisition

The Δ*feoB*′ mutation was predicted to be competent for siderophore-mediated iron acquisition. We confirmed that the Δ*feoB*′ mutant was capable of siderophore production using the universal Chrome Azurol S assay (CAS) [42] to assess levels of siderophore in supernatants of bacteria grown in iron-limiting che-CDM. The LVS Δ*fslA* strain lacking the siderophore synthetase gene [14] was used as a negative control. As shown in Figure 2C, the Δ*feoB*′ strain, like wild-type LVS, was capable of secreting siderophore when grown under iron-limitation. We tested siderophore-mediated iron transport capability using ^55^Fe uptake assays with purified siderophore incubated with ^55^Fe, as previously described [20], [19]. Optimal siderophore-mediated iron acquisition in LVS requires the siderophore receptor FslE [19] and therefore the Δ*fslE* mutant was used as a control in this assay. Wild-type LVS and the mutant Δ*feoB*′ and Δ*fslE* strains were grown in iron-limiting liquid che-CDM and were evaluated for the ability to transport ^55^Fe-bound siderophore. As shown in Figure 2D, the Δ*feoB*′ mutant was capable of acquiring siderophore-bound ^55^Fe at levels comparable to wild-type LVS, whereas the siderophore receptor mutant Δ*fslE* was defective in ^55^Fe-siderophore uptake. These findings demonstrated that Feo-mediated ferrous iron transport and ferric-siderophore iron acquisition work independently in LVS.

#### iv. The *ΔfeoB*′ strain, like the Δ*fslA* mutant is capable of intracellular growth within J774A.1 murine cells

**Figure 4:**
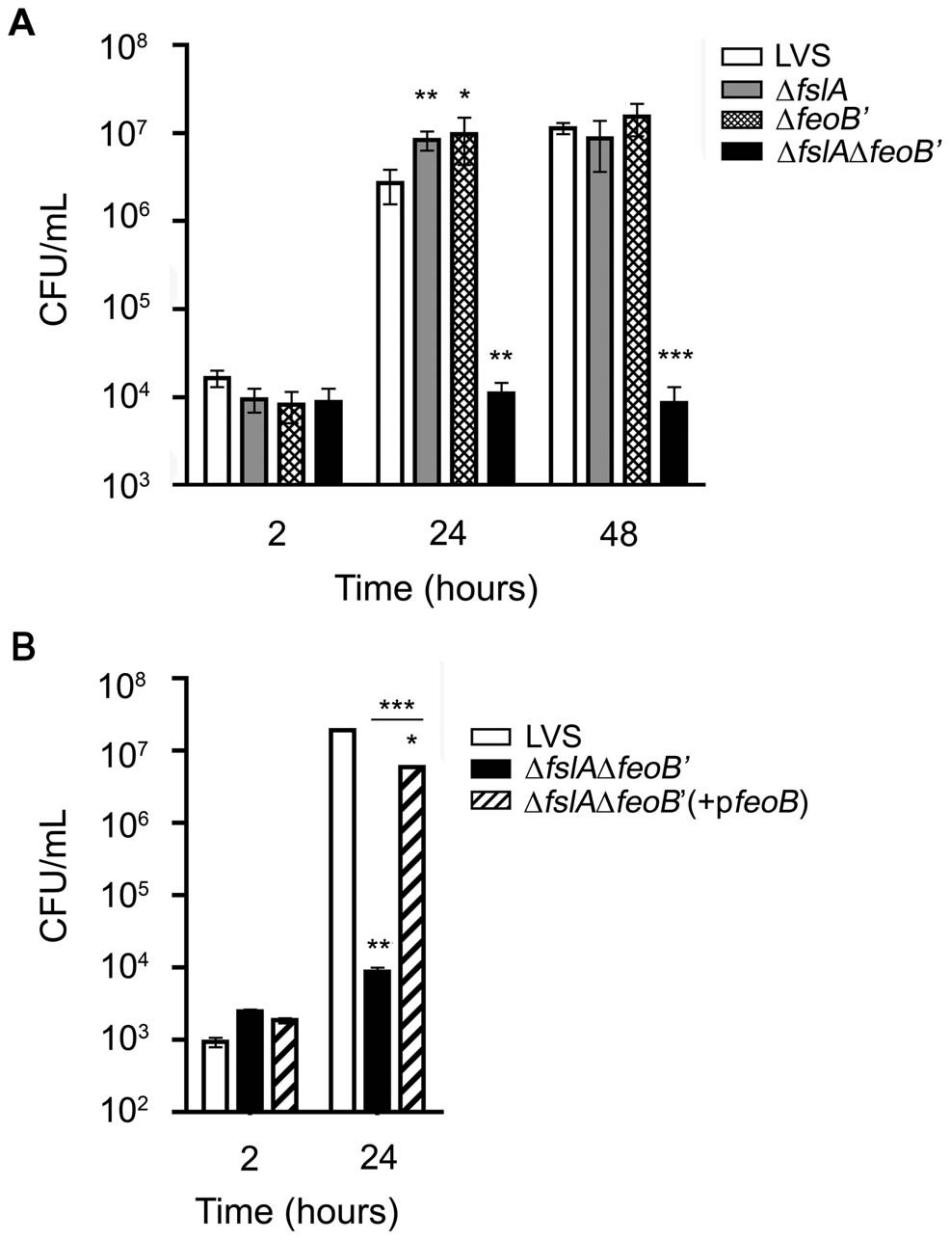
Intracellular replication of LVS iron acquisition mutants in J774A.1 macrophage-like cells. LVS and iron acquisition mutants were tested for intracellular replication in J774A.1 macrophages. Bacteria from frozen stocks were used at an MOI of 15 (A) and 5 (B). Infected cells were lysed at the times indicated and plated on MHA+ agar to determine CFU/mL. The Δ*fslA* Δ*feoB*′ mutant CFU/mL concentrations were determined on MHA+ agar topically supplemented with *F. tularensis* siderophore. A, LVS and the single and double deletion mutant CFU were compared at 2, 24 and 48 hours post infection. B, The Δ*fslA* Δ*feoB*′ and the *cis* complemented strains CFU were compared to wild-type LVS at 2 and 24 hours post infection. Values were expressed as the means ± S.D. Significance was calculated relative to LVS values. Values of the complemented strain were also compared to the mutant. * p<0.05, **p<0.01, ***p<0.001.

To test if ferrous iron uptake or siderophore-mediated iron acquisition is critical in supporting the intracellular lifecycle of *F. tularensis*, we compared the Δ*feoB*′ and Δ*fslA* strains to wild-type LVS for their ability to enter and replicate within the murine macrophage-like cell line J774A.1. LVS and single deletion mutants were able to enter J774A.1 cells at comparable numbers. At 24 hours post infection (Figure 4A) the single deletion mutants were able to replicate to numbers slightly higher than wild-type LVS. However, by 48 hours post-infection, all the strains grew to similar intracellular levels (Figure 4A). The ability of both the Δ*feoB*′ and Δ*fslA* strains to replicate within J774A.1 cells suggested that the *F. tularensis* ferric-siderophore and Feo-mediated ferrous iron acquisition systems were independent and could compensate for each other. Since bacteria typically possess multiple systems for iron acquisition, an alternative possibility was that an additional unidentified uptake system might compensate for the loss of either FeoB- or siderophore-mediated iron transport in LVS.

### Generation of the LVS Δ*fslA* Δ*feoB*′ mutant

To determine if an iron acquisition system was present in addition to the *fsl* and *feo* systems in LVS, a mutant carrying deletions in both *fslA* and *feoB* genes, LVS Δ*fslA* Δ*feoB*′, was generated. The process involved introduction of a suicide vector by electroporation into the Δ*feoB*′ strain to generate an in-frame deletion of *fslA*, as described previously [14]. Diagnostic PCR of initial isolates indicated mixed populations of the parent Δ*feoB*′ strain with the Δ*fslA* Δ*feoB*′ mutant. Attempts to select for single colonies of the Δ*fslA* Δ*feoB*′ mutant by plating on various agar media including CDM, cysteine heart agar plates with blood (CHAB), tryptic soy agar with cysteine (TSB/c) or MHA plates supplemented with FePPi, FeSO_4_, or horse blood were unsuccessful. The Δ*fslA* Δ*feoB*′ strain was also unable to grow in liquid culture media including CDM supplemented with FePPi or ferrous sulfate (FeSO_4_), Muller Hinton Broth, TSB/c and Brain Heart Infusion broth (BHI). We were able to finally isolate the strain using MHA agar topically supplemented with purified LVS siderophore. The purity of culture was confirmed on the basis of PCR analysis of genomic DNA.

#### i. The Δ*fslA* Δ*feoB*′ strain requires the addition of siderophore for growth

**Figure 5:**
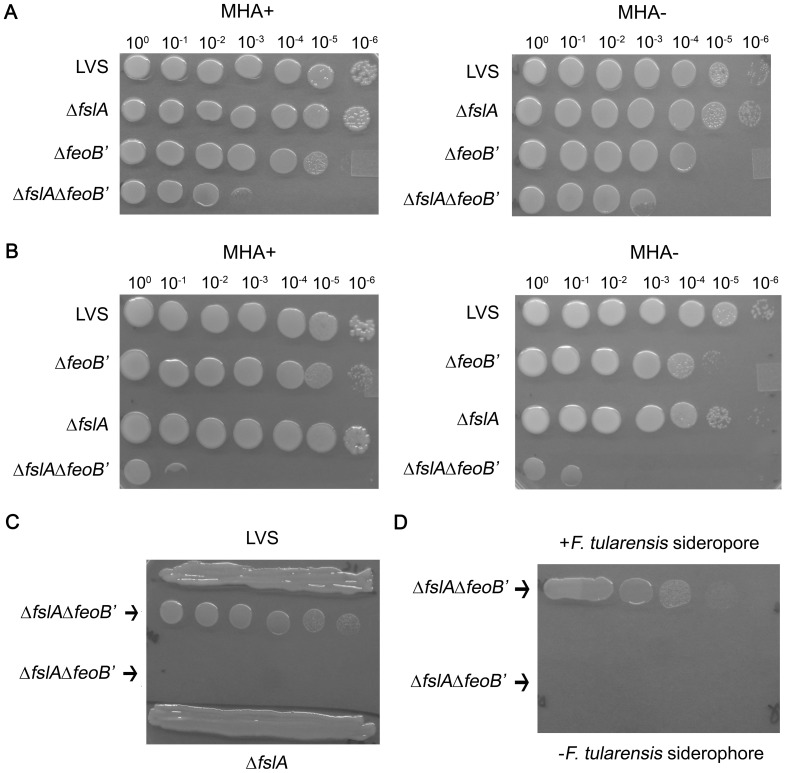
The Δ*fslA* Δ*feoB*′ strain requires *F. tularensis* siderophore for growth. A and B, LVS, Δ*fslA*, Δ*feoB*′, and Δ*fslA* Δ*feoB*′ strains grown on MHA+ agar plates were resuspended and brought to an OD_600_ of 1 in che-CDM. Strains were further serially diluted and spotted on MHA+ and MHA- plates. The order of Δ*fslA* and Δ*feoB*′ spots was switched in A and B. C, ten-fold serial dilutions of the mutant Δ*fslA* Δ*feoB*′ were spotted near dense streaks of LVS or Δ*fslA* strains on an MHA+ plate and grown for two days under aerobic 37°C conditions. D, ten-fold serial dilutions of the Δ*fslA* Δ*feoB*′ strain were spotted with (top) or without (bottom) 5 μL of 1.5 mM *F. tularensis* siderophore and grown for two days under aerobic 37°C conditions.

To demonstrate the phenotypic growth differences between wild-type LVS and the Δ*fslA*, Δ*feoB*′, and Δ*fslA* Δ*feoB*′ mutants, bacteria grown on MHA+ plates were collected and resuspended in CDM and ten-fold serial dilutions of all the strains were spotted on MHA+ and MHA- plates. The Δ*fslA* Δ*feoB*′ mutant was severely defective for growth on both MHA+ and MHA- plates but when spotted adjacent to the Δ*feoB*′ mutant, the double deletion mutant grew to the 10^−3^ dilution after 3 days (Figure 5A). The growth of the double deletion mutant was densest in the region proximal to the adjacent Δ*feoB*′ strain thus presenting a “half-moon” phenotype. This was consistent with the idea that the Δ*feoB*′ strain was secreting siderophore which helped to support growth of the Δ*fslA* Δ*feoB*′ mutant. To test this, the order of bacterial dilutions was switched (Figure 5B). When the siderophore-deficient Δ*fslA* strain was spotted adjacent to the Δ*fslA* Δ*feoB*′ mutant, the growth restriction of the Δ*fslA* Δ*feoB*′ mutant was more severe and was only able to reach the 10^−1^ dilution on both MHA+ and MHA- agar plates. The greater growth defect was likely due to the increased distance from the siderophore source (LVS Δ*feoB*′). Ten-fold serial dilutions of the Δ*fslA* Δ*feoB*′ mutant were spotted adjacent to a dense streak of siderophore producing LVS (Figure 5C, top) or siderophore deficient Δ*fslA* strain (bottom) on an MHA+ agar plate. After two days, only the Δ*fslA* Δ*feoB*′ strain spotted in the vicinity of LVS was able to grow out to single colonies. To confirm that growth was dependent only on exogenous siderophore, ten-fold dilutions of the Δ*fslA* Δ*feoB*′ strain were spotted on top of concentrated *F. tularensis* siderophore or on a region on the MHA+ plate without siderophore (Figure 5D). After two days, only the bacteria spotted over *F. tularensis* siderophore were able to grow. The Δ*fslA*′Δ*feoB*′ strain was also able to grow on CHAB and CDM agar if provided with *F. tularensis* siderophore (data not shown). Thus, the only way to potentiate growth of the double deletion mutant was in the presence of *F. tularensis* siderophore.

#### ii. The Δ*fslA* Δ*feoB*′ strain is defective for ferrous transport but is still capable of siderophore-bound ^55^Fe uptake

The Δ*fslA* Δ*feoB*′ strain growth defects were predicted to be due to the loss of both iron uptake systems. Its ability to grow on MHA+ with added *F. tularensis* siderophore suggested that this mutant was dependent on the ferric-siderophore acquisition system for survival. ^55^Fe uptake assays were used to analyze the iron transport capability of the Δ*fslA* Δ*feoB*′ strain in comparison to wild-type LVS and the Δ*fslA* and Δ*feoB*′ mutants. For this experiment, all the strains were grown on iron-rich plates similar to the double deletion mutant, followed by resuspension and a three-hour incubation with shaking in liquid che-CDM lacking iron. As expected, the Δ*fslA* Δ*feoB*′ mutant was defective for ^55^Fe ferrous iron uptake at both high and low ^55^Fe concentrations, just like the single deletion mutant Δ*feoB*′ mutant (Figure 3A and 3B). The Δ*fslA* Δ*feoB*′ strain complemented in *cis* with *feoB*, Δ*fslA* Δ*feoB*′ (*+*p*feoB*) regained the ability to acquire ferrous iron at both ferrous iron concentrations (Figure 3C and 3D).

The mutants were then examined for siderophore-mediated ferric iron transport. As might be expected following growth in iron-replete media, the LVS and single deletion mutants displayed a range of low, but detectable rates of ^55^Fe-siderophore transport that were consistent across experiments. The Δ*fslA* Δ*feoB*′ mutant however demonstrated a significantly greater rate of siderophore-bound ^55^Fe transport (Figure 3E), suggesting that the siderophore transport genes were more highly expressed in this strain. To explore this possibility, the levels of the FslE and FupA/B proteins (required for siderophore-iron transport) were tested by western blotting of bacterial lysates. GroEL was used as the loading control (Figure 3F). The *fslE* gene is Fur regulated and is only expressed under iron-limitation [17]. As expected, lysates from wild-type LVS and single deletion mutants grown on iron-replete MHA+ plates did not contain FslE. However, FslE was detectable in lysates prepared from the Δ*fslA* Δ*feoB*′ strain and the level resembled expression of LVS grown under iron-limiting conditions (Figure 3F). The observed expression of the siderophore receptor FslE suggested that the Δ*fslA* Δ*feoB*′ strain was in a state of iron starvation. FupA/B is not iron-regulated (unpublished data) and as expected, was produced in all strains at comparable levels (Figure 3F).

#### iii. The Δ*fslA* Δ*feoB*′ strain is attenuated for intracellular replication and for virulence in C57BL/6 mice

Agar plate studies of the Δ*fslA* Δ*feoB*′ strain described above showed that the double deletion mutant was only capable of growth with the addition of exogenous *F. tularensis* siderophore, indicating the absence of a third active iron acquisition mechanism in culture. Several genes important for *in vivo* survival and replication are induced following invasion of the macrophage [45] and we considered the possibility that these could include additional iron acquisition mechanisms. To test if an unidentified iron acquisition mechanism may be induced in the intracellular niche, the ability of the Δ*fslA* Δ*feoB*′ mutant to infect and replicate within J774A.1 cells was tested in comparison to LVS wild-type, Δ*fslA*, and Δ*feoB*′ strains (Figure 4A). All the strains, including the Δ*fslA* Δ*feoB*′ mutant, were able to infect the J774A.1 cells equally. The Δ*fslA* Δ*feoB*′ mutant, in contrast to the single deletion mutants Δ*fslA* and Δ*feoB*′, showed no increase in intracellular CFU at 24 and 48 hours post infection. This defect in intracellular replication was abrogated when the Δ*fslA* Δ*feoB*′ strain was complemented with the wild-type gene *feoB* (Figure 4B). Thus, there appeared to be no alternative iron acquisition system to support growth of the Δ*fslA* Δ*feoB*′ mutant in J774A.1 cells.

**Figure 6:**
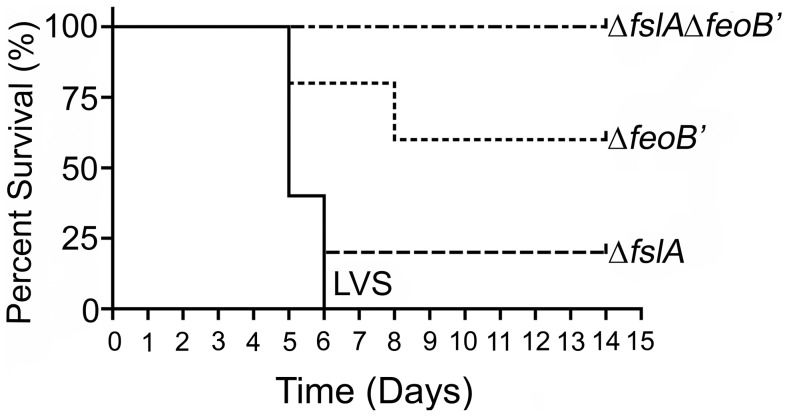
The Δ*fslA* Δ*feoB*′ strain is attenuated in virulence. 7-week-old C57BL/6 mice (5 per group) were infected by the intraperitoneal route with 1000 CFU of frozen stocks of bacterial strains. CFU administered were determined by plating on MHA+ with additional supplementation of *F. tularensis* siderophore for growth of the Δ*fslA* Δ*feoB*′ mutant. The difference in survival curves of LVS Δ*feoB*′ and LVS Δ*fslA* Δ*feoB*′ relative to LVS was statistically significant, p<0.05 and p<0.01, respectively. Survivors were challenged with 1000 CFU LVS at day 14 and all mice survived for greater than 14 days.

To test the impact of iron acquisition defects on virulence, we evaluated the bacterial mutants in a mouse model of tularemia. The number of LVS bacteria required to cause disease in the mouse model varies depending on the route of infection [9]. Infection with wild-type LVS at even a low dose (<10 CFU) by the intraperitoneal route (IP) has been demonstrated to produce a disease akin to human tularemia in mice [9]. Groups of C57BL/6 mice were infected with 1000 CFU of LVS and iron acquisition mutants by the IP route. Mice were monitored for two weeks (Figure 6). All mice infected with LVS (5/5) and the majority of mice infected with the Δ*fslA* strain (4/5) died within 6 days of infection. The Δ*feoB*′ mutant was partially attenuated for virulence, with 3/5 mice surviving the infection. All mice infected with the Δ*fslA*Δ*feoB*′ strain survived. Survivors of the initial infection were challenged with a lethal dose of LVS (1000 CFU by IP) to determine protection and all the mice survived up to 14 days, at which point the experiment was terminated. These experiments indicated that both siderophore-iron acquisition and ferrous iron acquisition contribute to virulence although ferrous iron acquisition likely played a more significant role. The attenuation of the double deletion mutant indicated that, as seen also in the intracellular replication assay, no alternative iron acquisition strategies existed to support *in vivo* survival of the bacteria.

## Discussion

This study comparing single and double deletion mutants in siderophore biosynthesis and ferrous iron uptake has established that the *F. tularensis* strain LVS possesses only two mechanisms for iron acquisition: the *fsl*-locus encoded ferric-siderophore and *feoB*-mediated ferrous iron acquisition systems. ^55^Fe uptake assays clearly demonstrated that the Δ*feoB*′ mutant is completely deficient in ferrous iron uptake, revealing the Feo system as the sole transporter of ferrous iron across the inner membrane of *F. tularensis*. However the Δ*feoB*′ mutant is capable of siderophore production and siderophore-mediated iron acquisition. The siderophore biosynthesis mutant Δ*fslA* is unable to synthesize siderophore [14] but is proficient at ferrous iron acquisition based on the ^55^Fe assays. The ^55^Fe assays have demonstrated that these two iron acquisition systems are specific to different forms of iron and function independently.

*Ex vivo* the Δ*feoB*′ and Δ*fslA* strains were capable of entering and replicating within J774A.1 macrophage-like cells to CFUs on par with and exceeding the wild-type LVS strain. These results suggest that while free iron is limited within mammalian cells, both ferric and ferrous iron sources are available to LVS within the cytoplasm of J774A.1 cells and that the *F. tularensis* siderophore and ferrous iron uptake mechanisms work in concert to provide enough iron for growth and replication of the pathogen. To determine if another iron acquisition system is available to LVS, a double deletion mutant was generated. The Δ*fslA* Δ*feoB*′ mutant could only be cultivated by providing *F. tularensis* siderophore on MHA+ plates. While the Δ*fslA* Δ*feoB*′ mutant was able to enter and survive in J774A.1 cells, it showed no increase in intracellular CFUs. This is similar to LVS mutants defective in purine [46] and guanine nucleotide biosynthesis [47], which are unable to replicate due to an inability to acquire necessary nutrients. The inability of the double deletion mutant to replicate intracellularly and its lack of virulence in mice demonstrates unequivocally that the ferric-siderophore and Feo systems are the only significant means of iron acquisition and are both necessary for full virulence in the mouse model of infection.

Intracellular growth capability may be influenced by differences in iron metabolism within different cell types. A strain lacking *feoB* was shown to have a deficient growth phenotype in epithelial and hepatic tissue culture cells [39]. However, macrophages are important for *Francisella* dissemination [10] and we confirmed by our study that a Δ*feoB*′ mutant is still capable of growing within macrophage-like J774 cells [39]. Loss of siderophore production as with the Δ*fslA* mutant in our study, or a Δ*fslC* mutant [39] does not affect the ability to replicate within a macrophage. Intra-macrophage growth is hampered only if both FeoB function and siderophore biosynthesis is disrupted.

The lack of another iron acquisition system in *F. tularensis* strain LVS demonstrates the effectiveness of the *F. tularensis* siderophore and Feo systems for survival and pathogenesis. Our analysis indicates that J774A.1 macrophage-like cells contain sufficient amounts of ferrous and ferric iron to satisfy the needs of LVS. The iron available within the cytoplasm is thought to include iron bound by host proteins such as ferritin and heme as well as within the transitional LIP. It has been shown that *F. tularensis* LVS co-localizes with transferrin receptor 1 (TfR1) during the initial stages of internalization and also promotes the host cell's expression of the transferrin receptor [48]. The TfR1 transferrin receptor pathway delivers iron into the cytoplasmic LIP through the action of the iron transporter Dmt1 and the reducing action of the Steap3 ferrireductase and thus makes iron available for *F. tularensis* in the cytoplasm [48]. It has also been suggested that *F. tularensis* siderophore is capable of removing iron from transferrin [49]. The ability to switch and/or simultaneously use both iron transport systems gives *F. tularensis* an advantage to survive within the iron-limiting environment of the host.

We found using an intraperitoneal infection model that the Δ*fslA* mutant was unaffected in virulence but the Δ*feoB*′ mutant was partially attenuated. In an intradermal model of infection however, a Δ*feoB* mutant did not demonstrate virulence attenuation following administration of a lethal dose, although differences in tissue burden were detected with a sublethal dose [39]. Both the route of infection and mouse genotype could lead to the differences in our results. We found that the double deletion mutant was avirulent following administration of CFUs corresponding to a lethal LVS dose. Interestingly, all mice in our study that survived the challenge with single Δ*feoB*′ or double deletion mutant were resistant to subsequent rechallenge with a lethal dose of LVS.

While the role of FeoA was not examined in our study, we anticipate that this predicted cytoplasmic protein could interact with FeoB as recently shown in the bacterial systems of *S*. Typhimurium [28], [29] and *Vibrio cholera* [50]. The Fur protein is known to regulate expression of iron acquisition systems and studies of an LVS Δ*fur* mutant (G. Ramakrishnan, unpublished data) have demonstrated that *feoB* is regulated by Fur. Since the Feo system is the sole inner membrane ferrous transport system in LVS, Fur and FeoA may also play significant roles in the FeoB-mediated transport of ferrous iron.

*F. tularensis* includes the less studied *mediasiatica* subspecies in addition to *tularensis* and *holarctica*, all of which are human pathogens and have reduced genomes in comparison to the near ancestral relative *F. novicida* within the same evolutionary clade [51]–[53]. *F. novicida* is not normally a human pathogen but is capable of intracellular replication in murine and human macrophages and can cause a tularemia-like disease in mice [54]. Interestingly, the *fsl* and *feo* genes are conserved among all of these isolates (www.patricbrc.org), suggesting that the iron acquisition systems may play an important role in supporting survival in the pathogenic intracellular milieu although it is possible that the more metabolically competent *F. novicida* may encode additional iron acquisition systems. Conservation of the *fsl* and *feo* iron acquisition systems does not necessarily suggest that both systems are equally utilized in different scenarios. Similar studies in the virulent *tularensis* subspecies may weigh the importance of both iron acquisition systems and their necessity for survival and virulence. Additionally, the ability of the LVS Δ*fslA* Δ*feoB*′ mutant to generate resistance to subsequent lethal LVS challenge suggests that a similar mutant may also be generated in the *tularensis* subspecies as an effective live vaccine strain.
